# Supplementary material for: Modeling glioblastoma heterogeneity as a dynamic network of cell states
Source: Mol Syst Biol. 2021 Sep 16;17(9):e10105. doi: 10.15252/msb.202010105 (PMC8444284; doi:10.15252/msb.202010105)
Supplement: Supplementary file 6 — Source Data for Figure 5 [file MSB-17-e10105-s004.zip › Figure5A_sourcedata/GSEA_3071/nosigdb_stateV.GseaPreranked.1622795159954/GAROFANO_IAVARONE_PPR_SUBTYPE.html]

Details for gene set GAROFANO\_IAVARONE\_PPR\_SUBTYPE[GSEA]

|  || Dataset | state43071 |
| Phenotype | NoPhenotypeAvailable |
| Upregulated in class | na\_pos |
| GeneSet | GAROFANO\_IAVARONE\_PPR\_SUBTYPE |
| Enrichment Score (ES) | 0.6543262 |
| Normalized Enrichment Score (NES) | 4.2989683 |
| Nominal p-value | 0.0 |
| FDR q-value | 0.0 |
| FWER p-Value | 0.0 |
Table: GSEA Results Summary

  

Fig 1: Enrichment plot: GAROFANO\_IAVARONE\_PPR\_SUBTYPE      
 Profile of the Running ES Score & Positions of GeneSet Members on the Rank Ordered List

  

| PROBE | GENE SYMBOL | GENE\_TITLE | RANK IN GENE LIST | RANK METRIC SCORE | RUNNING ES | CORE ENRICHMENT || 1 | HIST1H1B |  |  | 0 | 2.500 | 0.0081 | Yes |
| 2 | HIST1H4C |  |  | 1 | 2.442 | 0.0161 | Yes |
| 3 | ATAD2 |  |  | 2 | 2.214 | 0.0233 | Yes |
| 4 | ESCO2 |  |  | 3 | 2.193 | 0.0304 | Yes |
| 5 | FAM111B |  |  | 4 | 2.012 | 0.0369 | Yes |
| 6 | HMGB2 |  |  | 5 | 2.011 | 0.0435 | Yes |
| 7 | NUSAP1 |  |  | 6 | 2.001 | 0.0500 | Yes |
| 8 | PCNA |  |  | 10 | 1.908 | 0.0556 | Yes |
| 9 | TOP2A |  |  | 14 | 1.842 | 0.0610 | Yes |
| 10 | MCM4 |  |  | 17 | 1.815 | 0.0664 | Yes |
| 11 | NCAPG |  |  | 21 | 1.760 | 0.0716 | Yes |
| 12 | RAD51AP1 |  |  | 22 | 1.757 | 0.0773 | Yes |
| 13 | H2AFX |  |  | 26 | 1.725 | 0.0823 | Yes |
| 14 | CENPF |  |  | 28 | 1.705 | 0.0876 | Yes |
| 15 | TYMS |  |  | 30 | 1.683 | 0.0929 | Yes |
| 16 | HIST2H2AC |  |  | 31 | 1.681 | 0.0983 | Yes |
| 17 | RRM2 |  |  | 34 | 1.660 | 0.1033 | Yes |
| 18 | MYBL2 |  |  | 35 | 1.651 | 0.1087 | Yes |
| 19 | MKI67 |  |  | 45 | 1.598 | 0.1121 | Yes |
| 20 | BRCA1 |  |  | 46 | 1.592 | 0.1172 | Yes |
| 21 | HELLS |  |  | 48 | 1.588 | 0.1222 | Yes |
| 22 | XRCC2 |  |  | 50 | 1.574 | 0.1271 | Yes |
| 23 | KIF15 |  |  | 52 | 1.568 | 0.1320 | Yes |
| 24 | FOXM1 |  |  | 53 | 1.550 | 0.1370 | Yes |
| 25 | EZH2 |  |  | 55 | 1.534 | 0.1418 | Yes |
| 26 | GINS2 |  |  | 57 | 1.528 | 0.1466 | Yes |
| 27 | DTL |  |  | 60 | 1.503 | 0.1511 | Yes |
| 28 | GTSE1 |  |  | 66 | 1.491 | 0.1549 | Yes |
| 29 | E2F8 |  |  | 67 | 1.488 | 0.1597 | Yes |
| 30 | CDK1 |  |  | 69 | 1.488 | 0.1644 | Yes |
| 31 | SMC2 |  |  | 73 | 1.466 | 0.1685 | Yes |
| 32 | SPC25 |  |  | 76 | 1.442 | 0.1728 | Yes |
| 33 | CHAF1A |  |  | 77 | 1.441 | 0.1775 | Yes |
| 34 | UBE2T |  |  | 79 | 1.432 | 0.1819 | Yes |
| 35 | FANCD2 |  |  | 81 | 1.423 | 0.1864 | Yes |
| 36 | KLHL23 |  |  | 83 | 1.421 | 0.1908 | Yes |
| 37 | CLSPN |  |  | 84 | 1.417 | 0.1954 | Yes |
| 38 | MCM7 |  |  | 88 | 1.400 | 0.1993 | Yes |
| 39 | MCM10 |  |  | 92 | 1.389 | 0.2032 | Yes |
| 40 | CDCA2 |  |  | 96 | 1.382 | 0.2071 | Yes |
| 41 | RRM1 |  |  | 99 | 1.364 | 0.2112 | Yes |
| 42 | POLD3 |  |  | 111 | 1.340 | 0.2133 | Yes |
| 43 | KIFC1 |  |  | 113 | 1.338 | 0.2174 | Yes |
| 44 | CENPM |  |  | 114 | 1.337 | 0.2218 | Yes |
| 45 | TACC3 |  |  | 116 | 1.334 | 0.2259 | Yes |
| 46 | ZNF704 |  |  | 120 | 1.329 | 0.2296 | Yes |
| 47 | CDCA5 |  |  | 123 | 1.326 | 0.2335 | Yes |
| 48 | SMC4 |  |  | 127 | 1.320 | 0.2372 | Yes |
| 49 | BARD1 |  |  | 132 | 1.310 | 0.2406 | Yes |
| 50 | FEN1 |  |  | 139 | 1.296 | 0.2436 | Yes |
| 51 | SALL3 |  |  | 142 | 1.286 | 0.2474 | Yes |
| 52 | RTKN2 |  |  | 143 | 1.285 | 0.2516 | Yes |
| 53 | LIG1 |  |  | 145 | 1.284 | 0.2556 | Yes |
| 54 | GMNN |  |  | 146 | 1.280 | 0.2597 | Yes |
| 55 | NCAPD3 |  |  | 155 | 1.261 | 0.2622 | Yes |
| 56 | CCNF |  |  | 158 | 1.250 | 0.2659 | Yes |
| 57 | POLQ |  |  | 160 | 1.245 | 0.2697 | Yes |
| 58 | TMPO |  |  | 161 | 1.243 | 0.2737 | Yes |
| 59 | CENPK |  |  | 162 | 1.241 | 0.2778 | Yes |
| 60 | LMNB1 |  |  | 165 | 1.238 | 0.2814 | Yes |
| 61 | DNMT1 |  |  | 166 | 1.237 | 0.2854 | Yes |
| 62 | KIF23 |  |  | 167 | 1.235 | 0.2894 | Yes |
| 63 | NCAPH |  |  | 168 | 1.235 | 0.2934 | Yes |
| 64 | NASP |  |  | 180 | 1.212 | 0.2951 | Yes |
| 65 | DBF4 |  |  | 189 | 1.197 | 0.2974 | Yes |
| 66 | KIF4A |  |  | 191 | 1.193 | 0.3011 | Yes |
| 67 | NUF2 |  |  | 192 | 1.189 | 0.3049 | Yes |
| 68 | CDT1 |  |  | 194 | 1.185 | 0.3086 | Yes |
| 69 | PXMP2 |  |  | 195 | 1.180 | 0.3124 | Yes |
| 70 | FANCI |  |  | 196 | 1.179 | 0.3163 | Yes |
| 71 | FANCA |  |  | 199 | 1.173 | 0.3197 | Yes |
| 72 | ZNF43 |  |  | 201 | 1.169 | 0.3233 | Yes |
| 73 | ZWINT |  |  | 204 | 1.166 | 0.3267 | Yes |
| 74 | ASPM |  |  | 207 | 1.163 | 0.3300 | Yes |
| 75 | PRC1 |  |  | 211 | 1.161 | 0.3332 | Yes |
| 76 | KIF22 |  |  | 213 | 1.157 | 0.3367 | Yes |
| 77 | UBE2C |  |  | 214 | 1.154 | 0.3405 | Yes |
| 78 | DPF1 |  |  | 220 | 1.150 | 0.3432 | Yes |
| 79 | NCAPG2 |  |  | 225 | 1.141 | 0.3461 | Yes |
| 80 | CDC45 |  |  | 228 | 1.138 | 0.3494 | Yes |
| 81 | CDK2 |  |  | 233 | 1.134 | 0.3523 | Yes |
| 82 | ASF1B |  |  | 237 | 1.130 | 0.3554 | Yes |
| 83 | MCM2 |  |  | 238 | 1.130 | 0.3590 | Yes |
| 84 | RFC3 |  |  | 239 | 1.130 | 0.3627 | Yes |
| 85 | NDC80 |  |  | 240 | 1.129 | 0.3664 | Yes |
| 86 | MCM5 |  |  | 243 | 1.127 | 0.3696 | Yes |
| 87 | TPX2 |  |  | 248 | 1.122 | 0.3725 | Yes |
| 88 | BRIP1 |  |  | 249 | 1.121 | 0.3761 | Yes |
| 89 | SMC1A |  |  | 251 | 1.120 | 0.3795 | Yes |
| 90 | TIMELESS |  |  | 259 | 1.105 | 0.3817 | Yes |
| 91 | MAP2 |  |  | 262 | 1.100 | 0.3849 | Yes |
| 92 | KIF14 |  |  | 272 | 1.090 | 0.3866 | Yes |
| 93 | HAUS8 |  |  | 273 | 1.089 | 0.3901 | Yes |
| 94 | BRCA2 |  |  | 274 | 1.089 | 0.3937 | Yes |
| 95 | BUB1 |  |  | 277 | 1.084 | 0.3968 | Yes |
| 96 | MCM6 |  |  | 282 | 1.080 | 0.3995 | Yes |
| 97 | CKAP2L |  |  | 285 | 1.078 | 0.4026 | Yes |
| 98 | ADD2 |  |  | 286 | 1.078 | 0.4061 | Yes |
| 99 | HIRIP3 |  |  | 289 | 1.077 | 0.4092 | Yes |
| 100 | PRIM1 |  |  | 293 | 1.074 | 0.4121 | Yes |
| 101 | ECT2 |  |  | 295 | 1.071 | 0.4153 | Yes |
| 102 | AURKB |  |  | 297 | 1.068 | 0.4186 | Yes |
| 103 | ARHGAP11A |  |  | 303 | 1.064 | 0.4211 | Yes |
| 104 | STMN1 |  |  | 307 | 1.062 | 0.4239 | Yes |
| 105 | CCNA2 |  |  | 309 | 1.057 | 0.4271 | Yes |
| 106 | CKAP2 |  |  | 312 | 1.053 | 0.4302 | Yes |
| 107 | DUT |  |  | 314 | 1.050 | 0.4334 | Yes |
| 108 | CDC6 |  |  | 328 | 1.040 | 0.4341 | Yes |
| 109 | CDCA7 |  |  | 330 | 1.038 | 0.4373 | Yes |
| 110 | DDX11 |  |  | 331 | 1.038 | 0.4406 | Yes |
| 111 | RFC2 |  |  | 337 | 1.025 | 0.4430 | Yes |
| 112 | KNTC1 |  |  | 342 | 1.023 | 0.4455 | Yes |
| 113 | POLE |  |  | 344 | 1.023 | 0.4486 | Yes |
| 114 | WDR76 |  |  | 350 | 1.015 | 0.4509 | Yes |
| 115 | EXO1 |  |  | 354 | 1.010 | 0.4536 | Yes |
| 116 | RBL1 |  |  | 358 | 0.999 | 0.4562 | Yes |
| 117 | CDKN1B |  |  | 360 | 0.997 | 0.4592 | Yes |
| 118 | KIF11 |  |  | 369 | 0.982 | 0.4608 | Yes |
| 119 | DNAJC9 |  |  | 373 | 0.979 | 0.4634 | Yes |
| 120 | PARP1 |  |  | 374 | 0.978 | 0.4666 | Yes |
| 121 | DSN1 |  |  | 377 | 0.975 | 0.4693 | Yes |
| 122 | CASP8AP2 |  |  | 379 | 0.970 | 0.4723 | Yes |
| 123 | WDR62 |  |  | 384 | 0.966 | 0.4746 | Yes |
| 124 | GINS1 |  |  | 385 | 0.965 | 0.4777 | Yes |
| 125 | DEPDC1B |  |  | 387 | 0.963 | 0.4807 | Yes |
| 126 | HJURP |  |  | 406 | 0.945 | 0.4801 | Yes |
| 127 | MASTL |  |  | 407 | 0.945 | 0.4831 | Yes |
| 128 | RMI1 |  |  | 411 | 0.939 | 0.4856 | Yes |
| 129 | SPC24 |  |  | 414 | 0.938 | 0.4882 | Yes |
| 130 | NUDT1 |  |  | 417 | 0.936 | 0.4909 | Yes |
| 131 | MAD2L1 |  |  | 421 | 0.932 | 0.4933 | Yes |
| 132 | SAP30 |  |  | 424 | 0.930 | 0.4959 | Yes |
| 133 | DSCC1 |  |  | 427 | 0.926 | 0.4985 | Yes |
| 134 | KPNA2 |  |  | 435 | 0.919 | 0.5001 | Yes |
| 135 | CENPE |  |  | 437 | 0.918 | 0.5029 | Yes |
| 136 | LBR |  |  | 438 | 0.916 | 0.5058 | Yes |
| 137 | INCENP |  |  | 440 | 0.916 | 0.5086 | Yes |
| 138 | RACGAP1 |  |  | 443 | 0.912 | 0.5112 | Yes |
| 139 | CENPH |  |  | 448 | 0.908 | 0.5133 | Yes |
| 140 | STIL |  |  | 455 | 0.903 | 0.5150 | Yes |
| 141 | ZNF738 |  |  | 458 | 0.901 | 0.5175 | Yes |
| 142 | POLA1 |  |  | 463 | 0.900 | 0.5197 | Yes |
| 143 | HMGN2 |  |  | 466 | 0.900 | 0.5222 | Yes |
| 144 | POC1A |  |  | 467 | 0.896 | 0.5251 | Yes |
| 145 | DNA2 |  |  | 470 | 0.890 | 0.5276 | Yes |
| 146 | CENPJ |  |  | 479 | 0.885 | 0.5288 | Yes |
| 147 | MND1 |  |  | 480 | 0.885 | 0.5317 | Yes |
| 148 | SPAG5 |  |  | 481 | 0.884 | 0.5346 | Yes |
| 149 | BLM |  |  | 486 | 0.879 | 0.5366 | Yes |
| 150 | PKMYT1 |  |  | 506 | 0.859 | 0.5356 | Yes |
| 151 | ZNF519 |  |  | 508 | 0.857 | 0.5381 | Yes |
| 152 | UBE2S |  |  | 514 | 0.854 | 0.5399 | Yes |
| 153 | RAD51 |  |  | 522 | 0.851 | 0.5412 | Yes |
| 154 | PLK1 |  |  | 526 | 0.850 | 0.5434 | Yes |
| 155 | LMNB2 |  |  | 534 | 0.846 | 0.5447 | Yes |
| 156 | BUB1B |  |  | 535 | 0.846 | 0.5475 | Yes |
| 157 | GAB1 |  |  | 536 | 0.846 | 0.5502 | Yes |
| 158 | BIRC5 |  |  | 541 | 0.842 | 0.5522 | Yes |
| 159 | CEP152 |  |  | 547 | 0.834 | 0.5539 | Yes |
| 160 | ZNF107 |  |  | 552 | 0.830 | 0.5557 | Yes |
| 161 | TP73 |  |  | 571 | 0.818 | 0.5547 | Yes |
| 162 | E2F2 |  |  | 576 | 0.814 | 0.5566 | Yes |
| 163 | HMGB1 |  |  | 583 | 0.810 | 0.5580 | Yes |
| 164 | CENPP |  |  | 587 | 0.808 | 0.5600 | Yes |
| 165 | FUS |  |  | 590 | 0.806 | 0.5622 | Yes |
| 166 | TK1 |  |  | 591 | 0.806 | 0.5648 | Yes |
| 167 | MSH6 |  |  | 593 | 0.804 | 0.5672 | Yes |
| 168 | LCORL |  |  | 594 | 0.804 | 0.5699 | Yes |
| 169 | RBBP4 |  |  | 610 | 0.794 | 0.5694 | Yes |
| 170 | RFWD3 |  |  | 616 | 0.790 | 0.5709 | Yes |
| 171 | GEN1 |  |  | 619 | 0.789 | 0.5731 | Yes |
| 172 | OIP5 |  |  | 620 | 0.788 | 0.5757 | Yes |
| 173 | CENPA |  |  | 627 | 0.786 | 0.5770 | Yes |
| 174 | ERI2 |  |  | 628 | 0.784 | 0.5796 | Yes |
| 175 | CDC25A |  |  | 631 | 0.782 | 0.5817 | Yes |
| 176 | PIF1 |  |  | 632 | 0.782 | 0.5842 | Yes |
| 177 | HNRNPD |  |  | 659 | 0.769 | 0.5815 | Yes |
| 178 | SKA3 |  |  | 668 | 0.765 | 0.5823 | Yes |
| 179 | C18orf54 |  |  | 669 | 0.765 | 0.5848 | Yes |
| 180 | ZNF85 |  |  | 670 | 0.765 | 0.5873 | Yes |
| 181 | RECQL4 |  |  | 676 | 0.761 | 0.5887 | Yes |
| 182 | KIF18A |  |  | 679 | 0.759 | 0.5908 | Yes |
| 183 | FANCB |  |  | 681 | 0.759 | 0.5931 | Yes |
| 184 | CDC25C |  |  | 684 | 0.755 | 0.5951 | Yes |
| 185 | CENPO |  |  | 686 | 0.755 | 0.5974 | Yes |
| 186 | SOX11 |  |  | 690 | 0.751 | 0.5992 | Yes |
| 187 | MBNL3 |  |  | 692 | 0.750 | 0.6014 | Yes |
| 188 | KIF18B |  |  | 698 | 0.747 | 0.6028 | Yes |
| 189 | SMC3 |  |  | 708 | 0.743 | 0.6034 | Yes |
| 190 | FIGNL1 |  |  | 714 | 0.741 | 0.6048 | Yes |
| 191 | PLK4 |  |  | 720 | 0.739 | 0.6062 | Yes |
| 192 | ATAD5 |  |  | 721 | 0.739 | 0.6086 | Yes |
| 193 | ANP32E |  |  | 722 | 0.739 | 0.6110 | Yes |
| 194 | MAD2L2 |  |  | 724 | 0.736 | 0.6132 | Yes |
| 195 | CTCF |  |  | 729 | 0.731 | 0.6148 | Yes |
| 196 | ZNF273 |  |  | 731 | 0.729 | 0.6169 | Yes |
| 197 | CKAP5 |  |  | 752 | 0.723 | 0.6152 | Yes |
| 198 | CCDC150 |  |  | 773 | 0.713 | 0.6135 | Yes |
| 199 | CHEK1 |  |  | 779 | 0.709 | 0.6148 | Yes |
| 200 | CBX5 |  |  | 783 | 0.708 | 0.6165 | Yes |
| 201 | MAML2 |  |  | 784 | 0.708 | 0.6188 | Yes |
| 202 | RAD54L |  |  | 790 | 0.704 | 0.6200 | Yes |
| 203 | CEP78 |  |  | 791 | 0.704 | 0.6223 | Yes |
| 204 | DTYMK |  |  | 800 | 0.700 | 0.6230 | Yes |
| 205 | TRIP13 |  |  | 804 | 0.698 | 0.6246 | Yes |
| 206 | TRIB2 |  |  | 827 | 0.688 | 0.6224 | Yes |
| 207 | SP4 |  |  | 832 | 0.687 | 0.6238 | Yes |
| 208 | H2AFZ |  |  | 851 | 0.677 | 0.6224 | Yes |
| 209 | RNASEH2A |  |  | 859 | 0.674 | 0.6231 | Yes |
| 210 | SKA1 |  |  | 868 | 0.672 | 0.6237 | Yes |
| 211 | DBF4B |  |  | 870 | 0.671 | 0.6257 | Yes |
| 212 | ZNF92 |  |  | 871 | 0.670 | 0.6279 | Yes |
| 213 | CHEK2 |  |  | 882 | 0.667 | 0.6280 | Yes |
| 214 | BRD7 |  |  | 883 | 0.667 | 0.6302 | Yes |
| 215 | ING2 |  |  | 908 | 0.657 | 0.6274 | Yes |
| 216 | MELK |  |  | 915 | 0.653 | 0.6283 | Yes |
| 217 | NEIL3 |  |  | 919 | 0.651 | 0.6298 | Yes |
| 218 | PAXIP1 |  |  | 922 | 0.650 | 0.6315 | Yes |
| 219 | NAB1 |  |  | 929 | 0.648 | 0.6324 | Yes |
| 220 | H2AFV |  |  | 933 | 0.647 | 0.6339 | Yes |
| 221 | LIN54 |  |  | 938 | 0.646 | 0.6352 | Yes |
| 222 | KIAA1841 |  |  | 940 | 0.646 | 0.6371 | Yes |
| 223 | CENPN |  |  | 953 | 0.638 | 0.6367 | Yes |
| 224 | TMEM97 |  |  | 963 | 0.636 | 0.6370 | Yes |
| 225 | DOT1L |  |  | 977 | 0.628 | 0.6364 | Yes |
| 226 | HAUS1 |  |  | 987 | 0.624 | 0.6366 | Yes |
| 227 | ZNF93 |  |  | 988 | 0.624 | 0.6386 | Yes |
| 228 | POLD1 |  |  | 998 | 0.620 | 0.6388 | Yes |
| 229 | RANBP1 |  |  | 999 | 0.620 | 0.6408 | Yes |
| 230 | CASP2 |  |  | 1001 | 0.620 | 0.6426 | Yes |
| 231 | LSM4 |  |  | 1003 | 0.619 | 0.6444 | Yes |
| 232 | ZNF100 |  |  | 1005 | 0.619 | 0.6462 | Yes |
| 233 | TUBA1B |  |  | 1012 | 0.617 | 0.6470 | Yes |
| 234 | MNS1 |  |  | 1034 | 0.613 | 0.6448 | Yes |
| 235 | PAFAH1B3 |  |  | 1039 | 0.612 | 0.6459 | Yes |
| 236 | CDCA8 |  |  | 1043 | 0.610 | 0.6473 | Yes |
| 237 | ZWILCH |  |  | 1048 | 0.609 | 0.6485 | Yes |
| 238 | FAM76B |  |  | 1052 | 0.607 | 0.6498 | Yes |
| 239 | BAZ1B |  |  | 1072 | 0.601 | 0.6479 | Yes |
| 240 | MAZ |  |  | 1079 | 0.598 | 0.6487 | Yes |
| 241 | PGAP1 |  |  | 1085 | 0.596 | 0.6496 | Yes |
| 242 | RBMX |  |  | 1095 | 0.594 | 0.6497 | Yes |
| 243 | ZNF90 |  |  | 1101 | 0.592 | 0.6506 | Yes |
| 244 | CBX2 |  |  | 1104 | 0.591 | 0.6521 | Yes |
| 245 | MSH2 |  |  | 1107 | 0.590 | 0.6536 | Yes |
| 246 | ING1 |  |  | 1122 | 0.587 | 0.6527 | Yes |
| 247 | SPATA5 |  |  | 1139 | 0.581 | 0.6513 | Yes |
| 248 | HMGB3 |  |  | 1154 | 0.577 | 0.6504 | Yes |
| 249 | POLA2 |  |  | 1161 | 0.574 | 0.6510 | Yes |
| 250 | ZNF101 |  |  | 1162 | 0.574 | 0.6529 | Yes |
| 251 | EME1 |  |  | 1165 | 0.573 | 0.6543 | Yes |
| 252 | NUCKS1 |  |  | 1178 | 0.570 | 0.6537 | No |
| 253 | RIF1 |  |  | 1204 | 0.560 | 0.6505 | No |
| 254 | NSMCE4A |  |  | 1217 | 0.557 | 0.6499 | No |
| 255 | EXOSC8 |  |  | 1230 | 0.553 | 0.6492 | No |
| 256 | DGCR8 |  |  | 1246 | 0.547 | 0.6479 | No |
| 257 | CTDSPL2 |  |  | 1256 | 0.544 | 0.6479 | No |
| 258 | CEP135 |  |  | 1264 | 0.541 | 0.6482 | No |
| 259 | TOP2B |  |  | 1269 | 0.540 | 0.6492 | No |
| 260 | CREBBP |  |  | 1299 | 0.532 | 0.6450 | No |
| 261 | KIF2C |  |  | 1309 | 0.530 | 0.6449 | No |
| 262 | PTBP1 |  |  | 1311 | 0.530 | 0.6464 | No |
| 263 | CENPI |  |  | 1351 | 0.519 | 0.6402 | No |
| 264 | RAD54B |  |  | 1354 | 0.518 | 0.6415 | No |
| 265 | ICA1L |  |  | 1369 | 0.515 | 0.6403 | No |
| 266 | CCNB2 |  |  | 1373 | 0.513 | 0.6414 | No |
| 267 | LIN9 |  |  | 1376 | 0.512 | 0.6426 | No |
| 268 | ELAVL1 |  |  | 1391 | 0.508 | 0.6414 | No |
| 269 | TRAIP |  |  | 1402 | 0.504 | 0.6410 | No |
| 270 | TUBA1A |  |  | 1403 | 0.504 | 0.6427 | No |
| 271 | PHC1 |  |  | 1423 | 0.498 | 0.6404 | No |
| 272 | PKNOX1 |  |  | 1425 | 0.498 | 0.6418 | No |
| 273 | C1orf112 |  |  | 1437 | 0.496 | 0.6412 | No |
| 274 | EXOSC9 |  |  | 1472 | 0.487 | 0.6359 | No |
| 275 | CKLF |  |  | 1480 | 0.486 | 0.6361 | No |
| 276 | C2CD4C |  |  | 1481 | 0.486 | 0.6376 | No |
| 277 | DCLRE1C |  |  | 1497 | 0.480 | 0.6362 | No |
| 278 | CKS1B |  |  | 1502 | 0.479 | 0.6369 | No |
| 279 | C5orf34 |  |  | 1514 | 0.478 | 0.6362 | No |
| 280 | SUV39H2 |  |  | 1523 | 0.476 | 0.6361 | No |
| 281 | HNRNPAB |  |  | 1541 | 0.471 | 0.6342 | No |
| 282 | HNRNPU |  |  | 1548 | 0.470 | 0.6345 | No |
| 283 | SRRT |  |  | 1563 | 0.467 | 0.6332 | No |
| 284 | CCDC88A |  |  | 1564 | 0.466 | 0.6347 | No |
| 285 | RSBN1L |  |  | 1582 | 0.463 | 0.6328 | No |
| 286 | HAUS3 |  |  | 1589 | 0.461 | 0.6331 | No |
| 287 | POP7 |  |  | 1591 | 0.461 | 0.6343 | No |
| 288 | SASS6 |  |  | 1618 | 0.457 | 0.6306 | No |
| 289 | SSRP1 |  |  | 1634 | 0.451 | 0.6290 | No |
| 290 | ZNF678 |  |  | 1651 | 0.446 | 0.6272 | No |
| 291 | MEX3A |  |  | 1667 | 0.443 | 0.6256 | No |
| 292 | ZNF486 |  |  | 1671 | 0.442 | 0.6264 | No |
| 293 | PDE7A |  |  | 1684 | 0.439 | 0.6254 | No |
| 294 | PHF2 |  |  | 1685 | 0.439 | 0.6268 | No |
| 295 | NR2C2AP |  |  | 1690 | 0.438 | 0.6274 | No |
| 296 | ILF3 |  |  | 1698 | 0.436 | 0.6274 | No |
| 297 | ESPL1 |  |  | 1733 | 0.430 | 0.6219 | No |
| 298 | ZNF48 |  |  | 1738 | 0.429 | 0.6225 | No |
| 299 | SETMAR |  |  | 1746 | 0.428 | 0.6225 | No |
| 300 | HNRNPL |  |  | 1747 | 0.428 | 0.6238 | No |
| 301 | PLEKHG2 |  |  | 1795 | 0.419 | 0.6157 | No |
| 302 | DCTPP1 |  |  | 1796 | 0.418 | 0.6170 | No |
| 303 | TIA1 |  |  | 1802 | 0.417 | 0.6174 | No |
| 304 | VEZF1 |  |  | 1804 | 0.417 | 0.6185 | No |
| 305 | KBTBD2 |  |  | 1810 | 0.416 | 0.6189 | No |
| 306 | CDK14 |  |  | 1822 | 0.415 | 0.6180 | No |
| 307 | ING3 |  |  | 1844 | 0.411 | 0.6150 | No |
| 308 | NFATC3 |  |  | 1845 | 0.411 | 0.6164 | No |
| 309 | ZNF664 |  |  | 1862 | 0.408 | 0.6144 | No |
| 310 | KDM2B |  |  | 1881 | 0.406 | 0.6121 | No |
| 311 | PPP5C |  |  | 1894 | 0.405 | 0.6110 | No |
| 312 | ATG4D |  |  | 1906 | 0.403 | 0.6101 | No |
| 313 | CYTH2 |  |  | 1938 | 0.398 | 0.6051 | No |
| 314 | CENPW |  |  | 1959 | 0.395 | 0.6023 | No |
| 315 | ADNP2 |  |  | 1963 | 0.394 | 0.6030 | No |
| 316 | LSM5 |  |  | 1982 | 0.391 | 0.6006 | No |
| 317 | HNRNPA0 |  |  | 1983 | 0.391 | 0.6018 | No |
| 318 | MIER3 |  |  | 2011 | 0.386 | 0.5976 | No |
| 319 | ZIK1 |  |  | 2013 | 0.386 | 0.5987 | No |
| 320 | HCFC1 |  |  | 2030 | 0.383 | 0.5967 | No |
| 321 | ZNF431 |  |  | 2066 | 0.377 | 0.5908 | No |
| 322 | MTBP |  |  | 2070 | 0.377 | 0.5914 | No |
| 323 | XPA |  |  | 2079 | 0.376 | 0.5910 | No |
| 324 | ZNF117 |  |  | 2123 | 0.371 | 0.5835 | No |
| 325 | ATP9B |  |  | 2163 | 0.366 | 0.5767 | No |
| 326 | NUP205 |  |  | 2169 | 0.366 | 0.5769 | No |
| 327 | HNRNPR |  |  | 2175 | 0.365 | 0.5771 | No |
| 328 | PNRC2 |  |  | 2176 | 0.365 | 0.5783 | No |
| 329 | BUD13 |  |  | 2239 | 0.358 | 0.5668 | No |
| 330 | SENP1 |  |  | 2242 | 0.358 | 0.5676 | No |
| 331 | DHX9 |  |  | 2253 | 0.356 | 0.5667 | No |
| 332 | ATRIP |  |  | 2256 | 0.356 | 0.5675 | No |
| 333 | PDIK1L |  |  | 2275 | 0.353 | 0.5649 | No |
| 334 | SPEN |  |  | 2331 | 0.344 | 0.5549 | No |
| 335 | ZNF75A |  |  | 2374 | 0.339 | 0.5475 | No |
| 336 | ZNF689 |  |  | 2379 | 0.338 | 0.5478 | No |
| 337 | TMSB15B |  |  | 2386 | 0.337 | 0.5476 | No |
| 338 | KIF24 |  |  | 2422 | 0.332 | 0.5416 | No |
| 339 | ZNF829 |  |  | 2431 | 0.331 | 0.5411 | No |
| 340 | SKA2 |  |  | 2445 | 0.329 | 0.5395 | No |
| 341 | ZNF22 |  |  | 2452 | 0.328 | 0.5393 | No |
| 342 | RBM14 |  |  | 2453 | 0.327 | 0.5404 | No |
| 343 | ZNF530 |  |  | 2473 | 0.325 | 0.5376 | No |
| 344 | HNRNPA2B1 |  |  | 2523 | 0.320 | 0.5287 | No |
| 345 | C2orf80 |  |  | 2664 | 0.303 | 0.5012 | No |
| 346 | KLHL22 |  |  | 2726 | 0.297 | 0.4898 | No |
| 347 | SNRPA |  |  | 2738 | 0.296 | 0.4885 | No |
| 348 | AP4M1 |  |  | 2748 | 0.295 | 0.4877 | No |
| 349 | TRRAP |  |  | 2763 | 0.293 | 0.4858 | No |
| 350 | PALB2 |  |  | 2836 | 0.285 | 0.4721 | No |
| 351 | GINS3 |  |  | 2847 | 0.284 | 0.4710 | No |
| 352 | CBX3 |  |  | 2851 | 0.284 | 0.4713 | No |
| 353 | SAE1 |  |  | 2872 | 0.283 | 0.4681 | No |
| 354 | C19orf57 |  |  | 2920 | 0.277 | 0.4595 | No |
| 355 | PHACTR4 |  |  | 2964 | 0.273 | 0.4516 | No |
| 356 | NIF3L1 |  |  | 2969 | 0.272 | 0.4517 | No |
| 357 | CEP72 |  |  | 2974 | 0.272 | 0.4518 | No |
| 358 | DHX15 |  |  | 3022 | 0.267 | 0.4431 | No |
| 359 | HNRNPH1 |  |  | 3026 | 0.266 | 0.4434 | No |
| 360 | CHAF1B |  |  | 3047 | 0.264 | 0.4402 | No |
| 361 | PBX4 |  |  | 3049 | 0.264 | 0.4408 | No |
| 362 | TNFAIP8L1 |  |  | 3081 | 0.261 | 0.4354 | No |
| 363 | NRF1 |  |  | 3093 | 0.261 | 0.4340 | No |
| 364 | DUSP28 |  |  | 3096 | 0.260 | 0.4344 | No |
| 365 | FAM72D |  |  | 3107 | 0.260 | 0.4332 | No |
| 366 | ZRANB3 |  |  | 3156 | 0.255 | 0.4243 | No |
| 367 | DUSP12 |  |  | 3266 | -0.261 | 0.4030 | No |
| 368 | ASXL3 |  |  | 3440 | -0.288 | 0.3688 | No |
| 369 | MRPS17 |  |  | 3610 | -0.317 | 0.3355 | No |
| 370 | MYLK2 |  |  | 3841 | -0.357 | 0.2900 | No |
| 371 | RP9 |  |  | 3887 | -0.365 | 0.2820 | No |
| 372 | ZNF593 |  |  | 4487 | -0.534 | 0.1621 | No |
| 373 | MRTO4 |  |  | 4572 | -0.574 | 0.1469 | No |
Table: GSEA details [plain text format]

  

Fig 2: GAROFANO\_IAVARONE\_PPR\_SUBTYPE: Random ES distribution      
 Gene set null distribution of ES for **GAROFANO\_IAVARONE\_PPR\_SUBTYPE**
